# A novel bladder phenotype in junctional epidermolysis bullosa: a case report

**DOI:** 10.3389/fped.2025.1555599

**Published:** 2025-08-21

**Authors:** Qingbao He, Meng Gui, Hao Wang, Lei Zhang

**Affiliations:** ^1^ Department of Minimally Invasive Urological Surgery, Children's Hospital Affiliated to Shandong University, Jinan, China; ^2^ Department of Minimally Invasive Urological Surgery, Jinan Children's Hospital, Jinan, China

**Keywords:** junctional epidermolysis bullosa, ITGB4 mutation, bladder involvement, papillomatous bladder thickening, follicular mucosal changes, rare disease

## Abstract

**Background:**

Junctional epidermolysis bullosa (JEB) is a rare inherited blistering disorder, and its urological spectrum remains poorly defined.

**Case Presentation:**

A 19-month-old boy carrying compound heterozygous *ITGB4* mutations (p.R252C, p.P305l) had a 17-month history of intermittent voiding. Ultrasound demonstrated focal papillomatous bladder-wall thickening, and cystoscopy showed scattered follicular mucosal changes without masses. Biopsies revealed mild oedema and chronic lymphocytic inflammation; no malignancy. Urine cultures were negative.

**Conclusion:**

This case broadens the reported urological spectrum of ITGB4-related JEB by illustrating a papillomatous–follicular bladder phenotype. Early urological evaluation in patients with JEB presenting with unexplained urinary symptoms may facilitate timely, targeted management and help prevent chronic complications.

## Introduction

Epidermolysis bullosa (EB) comprises a group of inherited mechanobullous disorders with an overall incidence of ≈1: 50,000 live births, of which junctional EB (JEB) accounts for 5%–10% (1, 2). According to the 2020 consensus reclassification (3), EB is subdivided into EB simplex, JEB, dystrophic EB and Kindler EB, each defined by the ultrastructural level of tissue cleavage and its causative genes. In addition to ITGB4-related JEB, bladder involvement has also been reported in JEB caused by laminin-332 gene variants (LAMA3, LAMB3, LAMC2), manifesting as urethral strictures, meatal stenosis or severe inflammatory cystitis (4, 5). JEB involves tissue separation at the lamina lucida within the basement membrane zone (6). It typically arises from mutations in genes encoding hemidesmosome components or associated integrins, compromising dermal-epidermal adhesion and causing widespread skin fragility, mucosal involvement, and multisystem complications (1).

The *ITGB4* gene, encoding the β4 subunit of the α6β4 integrin complex (a vital hemidesmosome component anchoring basal keratinocytes), is particularly significant (7). *ITGB4* mutations are classically linked to JEB with pyloric atresia (JEB-PA) (7), but phenotypic variability exists. *ITGB4* mutations also occur in patients with JEB without pyloric atresia or with milder/late-onset skin issues alongside significant extracutaneous involvement, including severe uropathy (8).

Urological complications occur in various EB forms, and patients with JEB are particularly susceptible. Documented issues include meatal (urethral) stenosis (up to 11.6% in JEB-Herlitz), urinary retention (9.3%), hydronephrosis (7.0%), bladder-wall hypertrophy (4.6%), and cystitis (9). The urothelium, like epidermis, relies on robust cell-matrix adhesion; α6β4 integrin disruptions can predispose it to damage (10). While skin and gastrointestinal JEB manifestations are well-documented, and general bladder pathologies (mucosal blistering, wall thickening, fibrosis, polypoid masses) are anecdotally noted in EB (4), specific bladder phenotypes linked to *ITGB4* mutations are largely uncharacterized.

We report a 19-month-old boy with junctional epidermolysis bullosa carrying compound-heterozygous ITGB4 variants who fulfils the diagnostic criteria for JEB with pyloric atresia (JEB-PA); the pyloric atresia was surgically corrected in the neonatal period. Here we present the clinical, imaging and genetic findings of this case and discuss their implications. We discuss the potential pathophysiological relevance of these changes, their relationship to the underlying ITGB4 deficiency, and how they further broaden the recognised genitourinary spectrum of ITGB4-related disease.

## Case presentation

### Patient history and clinical findings

**Figure 1:**
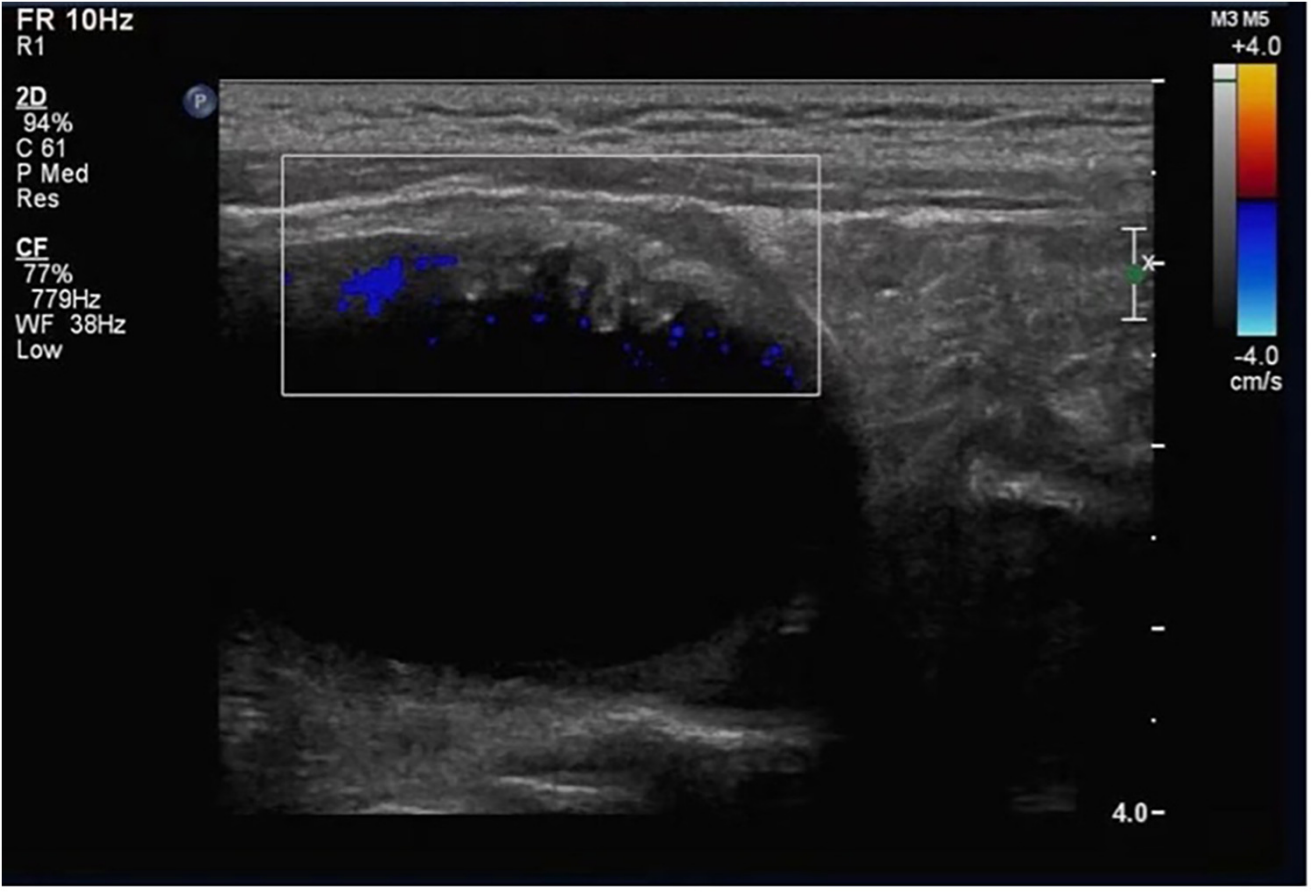
Ultrasound findings of the bladder wall. Localized papillomatous thickening of the bladder wall protruding into the bladder lumen, with visible vascular flow signal (color Doppler).

Skin fragility had been mild and episodic since birth; consequently, a definitive diagnosis of epidermolysis bullosa was not established until comprehensive genetic testing at 19 months of age. A 19-month-old boy presented with a 17-month history of difficult, interrupted urine streams noted by caregivers, accompanied by occasional discomfort. Congenital pyloric atresia was corrected surgically neonatally. At 2 months, he developed intermittent urinary difficulty with white flocculent material in his urine. A basic urinalysis at that age showed mild pyuria and hematuria; urine culture and crystal analysis were not performed. Antibiotics provided temporary relief, but episodes recurred. An ultrasound at 6 months revealed bilateral ureteral dilation; voiding cystourethrography showed no vesicoureteral reflux. Ureteral dilation resolved by 12 months. At 19 months, ultrasound revealed a focal bladder wall thickening (∼0.7 cm) that protruded into the lumen, exhibiting a papillomatous appearance (Figure 1).

He is the son of nonconsanguineous, asymptomatic parents; his older sister is unaffected. Physical examination showed partial nail loss/thickening, occasional subungual blistering, and multiple oral mucosal ulcers, consistent with EB. Genital/external urinary exams were normal. Cardiorespiratory and abdominal examinations were unremarkable.

### Laboratory tests

Urinalysis performed at 19 months revealed mild hematuria (red blood cells 306.5 cells µl^−1^) and pyuria (white blood cells 12.6 cells µl^−1^), with trace proteinuria; urine culture was negative.

### Imaging

Bladder ultrasound showed localized wall thickening protruding into the lumen, with a papillomatous appearance (Figure 1). Color Doppler confirmed vascular flow.

### Cystoscopy and histopathology

**Figure 2:**
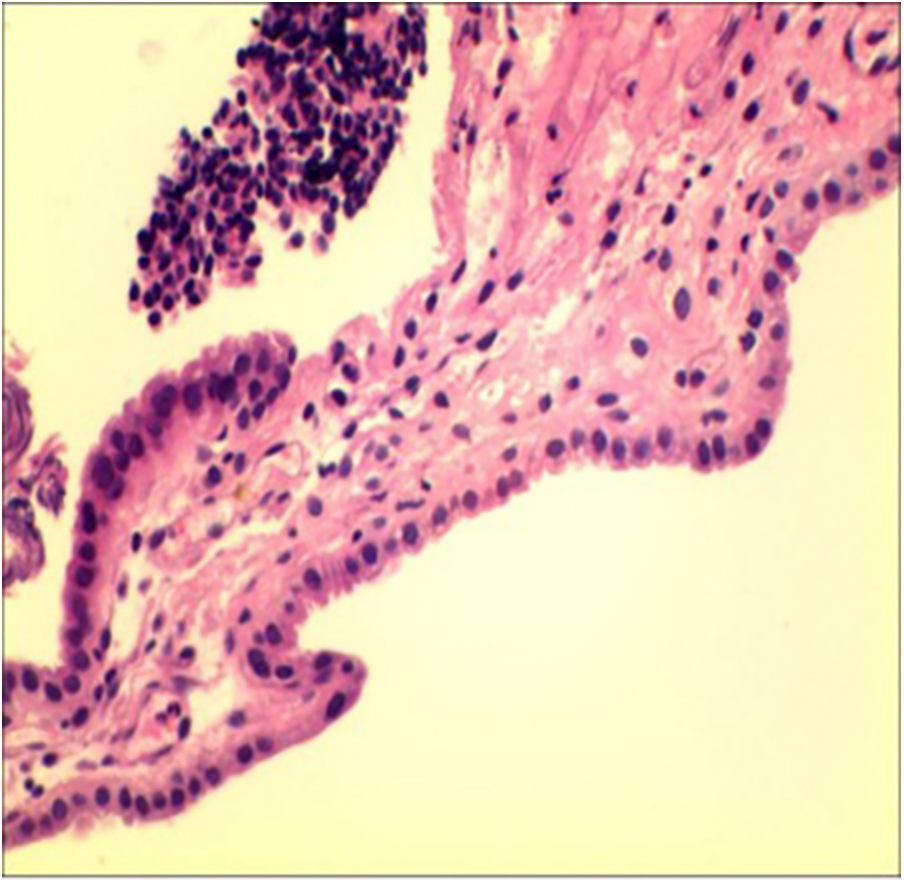
Histopathological examination of the bladder dome biopsy. Mild inflammation is observed, with edema and lymphocytic infiltration.

Cystoscopy revealed scattered, raised lesions exhibiting follicular mucosal changes on the bladder mucosa. Surrounding mucosa appeared pale and edematous. No solid masses were identified (Supplementary Figure 1). These projections were distinct from typical diffuse inflammatory changes. Bladder dome biopsies showed mild edema and chronic lymphocytic inflammatory infiltrate with vascular congestion. The urothelium was largely intact and no malignant cells were identified (Figure 2). Immunohistochemistry: Cytokeratin (+), Vimentin (+), PAX-8 (+), PAX-2 (+), few CyclinD1 (+) cells, SMA (−), BCL-2 (−), Ki67 <1%. Findings were consistent with chronic mucosal inflammation and no neoplasia; viral immunostains for human papillomavirus (p16) and polyomavirus SV40 were not performed owing to limited tissue.

### Genetic testing

**Figure 3:**
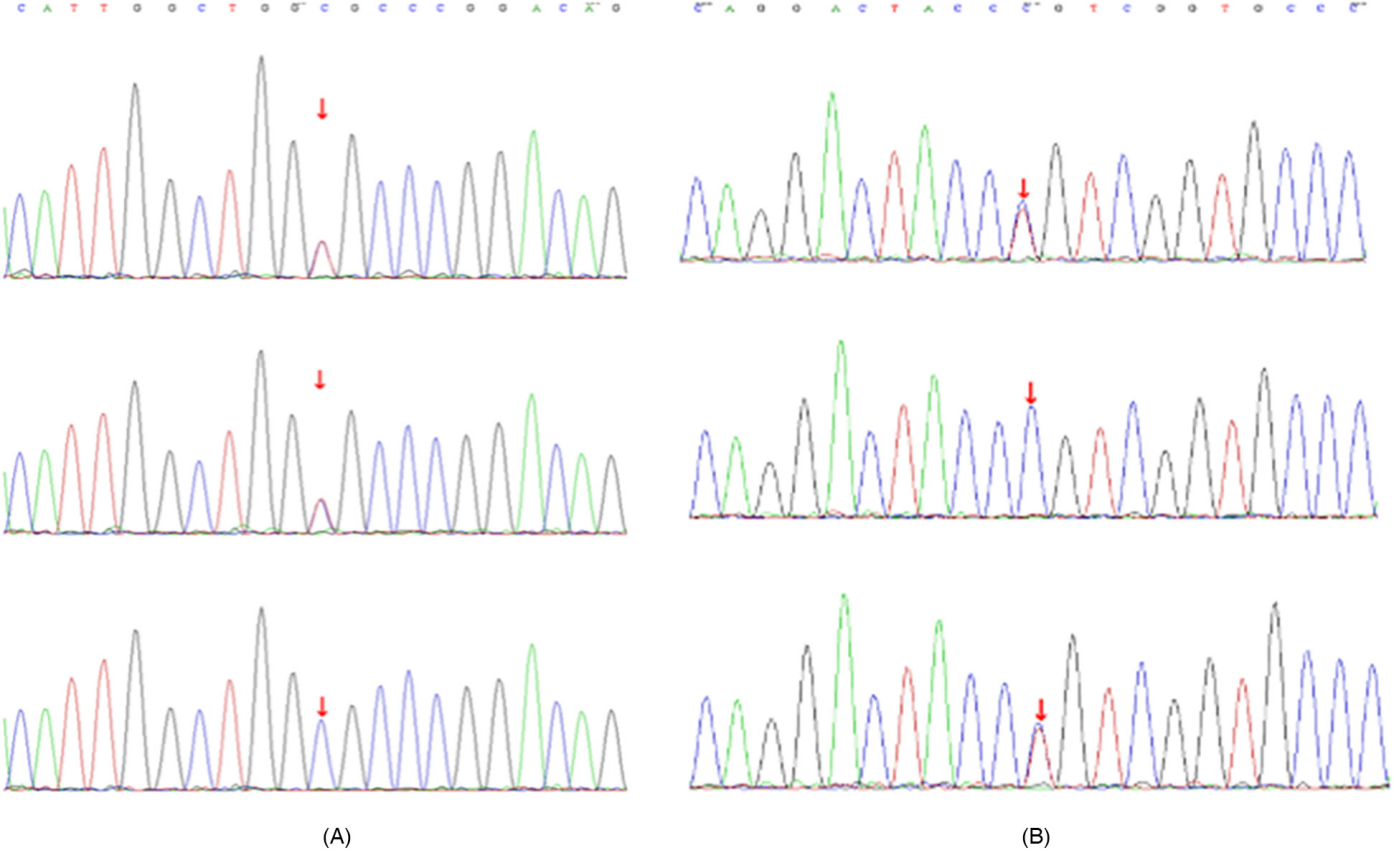
Sanger sequencing results of ITGB4 mutations. **(A)** Sanger sequencing showing the ITGB4 c.754C>T (p.R252C) mutation in the proband (top panel), father (middle panel), and mother (bottom panel). **(B)** Sanger sequencing showing the ITGB4 c.914C>T (p.P305l) mutation in the proband (top panel), father (middle panel), and mother (bottom panel).

Genetic analysis identified compound heterozygous ITGB4 mutations [c.754C>T (p.R252C) and c.914C>T (p.P305l)] (Supplementary Figure 2). Each mutation was inherited from an asymptomatic parent, consistent with autosomal recessive inheritance (Figures 3A,B; Supplementary Figures 3A,B). Bioinformatic tools (SIFT, PolyPhen-2, MutationTaster) suggested these variants were likely damaging. According to the American College of Medical Genetics and Genomics/Association for Molecular Pathology (ACMG/AMP) criteria, c.754C>T (p.Arg252Cys) meets evidence codes PVS1, PM1, PM2 and PP3 and is therefore classified as pathogenic, whereas c.914C>T (p.Pro305Leu) meets PM2 and PP3 and is classified as likely pathogenic. Both variants were queried against multiple population and clinical databases. They are absent from gnomAD v3.1 and v4.0 (allele count = 0). c.754C>T (p.Arg252Cys) is recorded in ClinVar and HGMD as pathogenic, whereas c.914C>T (p.Pro305Leu) is not listed in either database. The complete absence of population frequency, together with existing clinical annotations, further supports their pathogenicity. In-silico pathogenicity was further assessed with SIFT v6.2 (J. Craig Venter Institute, Rockville, USA); PolyPhen-2 v2.2 (Harvard University, Cambridge, USA); and MutationTaster (Charité—Universitätsmedizin Berlin, Germany). Structural modelling suggested p.Arg252Cys might disrupt hydrogen bonds, destabilising the integrin α6β4 complex, and p.Pro305Leu might affect protein flexibility.

**Figure 4:**
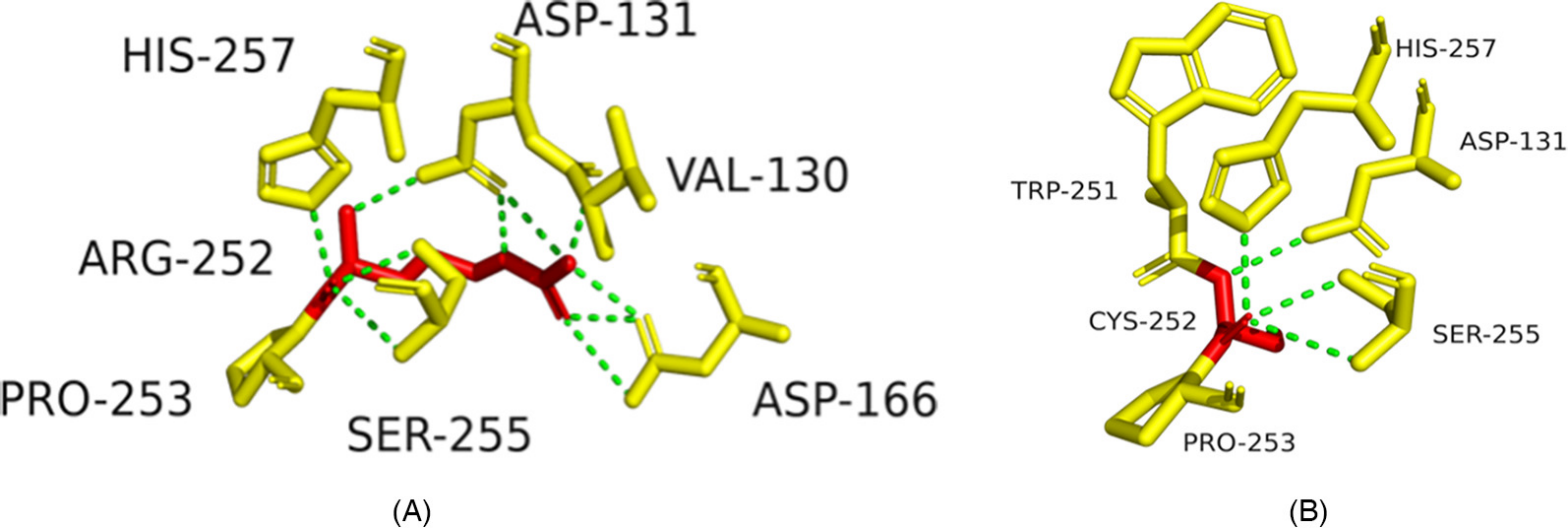
Structural impact of ITGB4 c.754C>T (p.R252C) mutation. **(A)** Wild-type ITGB4 structure showing key amino acid interactions. **(B)** Mutant ITGB4 structure with c.754C>T substitution, showing loss of hydrogen bonds.

**Figure 5:**
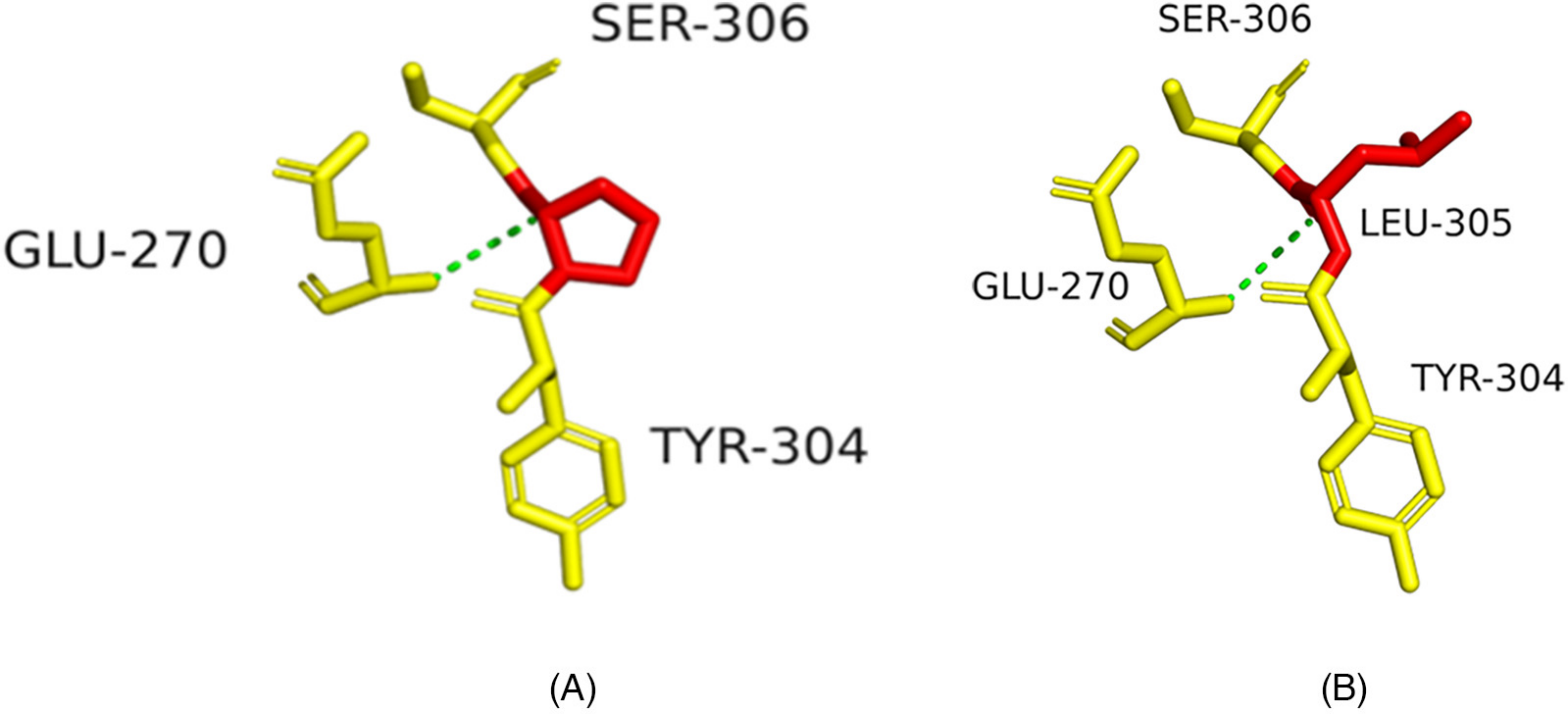
Structural impact of ITGB4 c.914C>T (p.P305l) mutation. **(A)** Wild-type ITGB4 structure showing key amino acid interactions. **(B)** Mutant ITGB4 structure with c.914C>T substitution, showing structural changes at LEU-305.

Structural modeling suggested p.R252C might disrupt hydrogen bonds, destabilizing the integrin α6β4 complex (Figures 4A,B), and p.P305l might affect protein flexibility (Figures 5A,B).

## Discussion

This report describes a patient with JEB who developed a previously undescribed papillomatous–follicular bladder phenotype associated with compound heterozygous ITGB4 variants. These findings expand the current understanding of urological manifestations in JEB. Similar papillomatous changes, described as “papilla bullosa”, were reported in an 11-year-old girl (parental origin documented) with ITGB4-related JEB by Ilmiers et al. in 2016 (11). In addition, phenotypic heterogeneity associated with β4-integrin mutations has been highlighted and commented on by Bruckner-Tuderman in an editorial comparing two siblings with divergent JEB manifestations (12).

### Contextualization with existing literature

Urological involvement in JEB is known. Previous reports focus on urethral strictures, hydronephrosis, urinary retention, general bladder wall hypertrophy, and cystitis (9). General bladder pathology in EB includes mucosal blistering, diffuse wall thickening, fibrosis, and occasional polypoid masses (4). A recent systematic review summarised 13 epidermolysis bullosa cases with vesicular or papillomatous cystitis, underscoring bladder mucosal vulnerability (13). The current case's papillomatous protrusion and accompanying follicular mucosal changes are morphologically distinct. Whereas “wall thickening” is a non-specific descriptor, a papillomatous projection denotes an organised outgrowth rather than simple hypertrophy. The follicular mucosal changes likely represent small, lymphoid-aggregate–related lesions that differ from larger polypoid formations (4). To our knowledge, this combination has not previously been described in the JEB literature.

### Significance of *ITGB4* mutations (p.R252c and p.P305l)

Compound-heterozygous ITGB4 variants p.R252C and p.P305l are central to this case. ITGB4 encodes the β4-integrin subunit, a key component of hemidesmosomes in epithelia such as the urothelium (6). Alterations at residue Arg252 are particularly noteworthy: Ellis et al. reported a patient with p.R252l who had mild cutaneous JEB-PA but severe obstructive uropathy (8), and Dang et al. documented a lethal JEB-PA neonate with p.R252C (14). These observations suggest that residue R252 is critical and that its alteration predisposes carriers to urological complications. Similar ITGB4-PA cases without bladder involvement have been reported (15). Reitelman et al. described an ITGB4-mutant patient with severe ureteric obstruction but no bladder lesions, further illustrating phenotypic variability (16). However, the specific “stalactite-like” bladder-wall thickening and “follicular” mucosal changes documented in our patient were not reported in those cases (8, 14). Ellis et al.'s patient had bladder-wall haemorrhage/blistering (8, 10) reported exuberant, de-epithelialised bladder mucosa in a child carrying different ITGB4 variants without pyloric atresia. This highlights that although R252 mutations are linked to uropathy, our patient's bladder manifestation is distinct. The p.P305l variant, far less characterised, was bio-informatically predicted as damaging and may further impair α6β4-integrin function. Although pyloric atresia defines the JEB-PA subtype, bladder involvement has been documented only sporadically. The coexistence of surgically corrected PA and a papillomatous–follicular bladder phenotype in our patient broadens the recognised genitourinary spectrum of ITGB4-related disease and underscores the need for systematic urological surveillance in JEB-PA.

Table 1 compares urological phenotypes.

**Table 1:** Comparison of urological phenotypes in reported cases of JEB with ITGB4 mutations^a^.

Reference (study/authors, year)	Patient age/sex	*ITGB4* mutations (specific variants)	Pyloric atresia (PA)	Key skin phenotype	Key urological/bladder findings	Outcome/severity
Current case (present report)	19-month-old M	c.754C>T (p.R252C) & c.914C>T (p.P305l) (Comp Het)	Present (Corrected)	Nail dystrophy, oral ulcers, intermittent skin blisters	Papillomatous bladder wall thickening, follicular mucosal changes; mild hematuria/pyuria; chronic inflammation	Ongoing, managed
Ellis et al. (2021) (8)	6-year-old M	p.R252l & 3793+1G>A (Comp Het)	Present	Mild cutaneous EB (blistering resolved early)	Severe obstructive uropathy; bladder wall hemorrhage/blistering; bilateral ureteric reflux; CKD stage 2	Nonlethal, severe uropathy
Dang et al. (2008) (14)	Neonate (d. 4 m)	658delC (PTC) & p.R252C (Comp Het)	Present	Severe skin blistering, erosions	Not detailed for bladder morphology	Lethal
Mattioli et al. (2022) (10)	10-year-old M	c.320G>C (p.Arg107Pro) & c.542C>T (p.Pro181Leu) (Comp Het)	Absent	Minimal, late-onset skin fragility (from age 6)	Severe obstructive uropathy; thickened bladder wall; exuberant/disepithelialized bladder mucosa; hydroureteronephrosis	Severe uropathy, ileal neo-bladder
Reitelman et al. (2023) (16)	8- year-old F	c.1234A>G; c.2345dup	Absent	Mild skin fragility	Severe ureteric obstruction; no bladder lesion	Managed

^a^ RBC, red blood cells; WBC, white blood cells; pyuria, elevated WBC in urine.

This comparison shows that while *ITGB4* mutations (especially at R252) link to severe uropathy, our patient's specific bladder phenotypes appear novel.

### Mechanistic insights

The novel phenotypes most plausibly arise from impaired urothelial integrity caused by dysfunctional α6β4 integrin. The ITGB4 variants (p.R252C, p.P305l) are predicted to destabilise the integrin complex, rendering the bladder mucosa vulnerable to mechanical stress, chronic inflammation and recurrent sub-clinical injury. Repeated insults may induce dysregulated repair, leading to chronic oedema, inflammatory infiltration and aberrant remodelling. The observed papillomatous projections could represent focal fibromuscular hyperplasia or organised granulation tissue, whereas the follicular mucosal changes suggest reactive lymphoid hyperplasia. Although urine cultures were negative, occult low-grade colonisation or sterile inflammation cannot be excluded. The widespread expression of α6β4 integrin in urogenital epithelium offers a biologically plausible link between integrin dysfunction, mucosal fragility and these secondary alterations (4).

### Clinical implications

Recognizing these novel bladder phenotypes in patients with JEB, especially with *ITGB4* mutations, may aid earlier diagnosis and management. This case reinforces recommendations for systematic urological evaluation (ultrasound, possibly cystoscopy) in patients with JEB with urinary symptoms or unexplained bladder anomalies (13, 17). Early identification could allow timely interventions to mitigate chronic irritation and prevent progressive dysfunction. A multidisciplinary approach is paramount.

### Future directions

1. Verification of Phenotypic Specificity
  Cohort studies or case series are needed to determine if these bladder changes are specific to certain JEB genotypes (e.g., *ITGB4* p.R252C) or broader manifestations.
2. Molecular Mechanism Studies
  Investigating how *ITGB4* mutations lead to these specific bladder phenotypes using patient-derived cells or organoids could identify dysregulated pathways or biomarkers.
3. Exploration of Gene Therapy
  Advances in gene therapy for EB, such as Beremagene geperpavec (B-VEC) for dystrophic EB and ex vivo LAMB3 correction for JEB (18), provide a rationale for developing ITGB4-targeting strategies, in line with recent insights into ITGB4 genetics and management (19). These approaches could potentially mitigate both cutaneous and systemic complications, including the bladder phenotype described here.

### Limitations

This single case report's findings may not be generalizable. Long-term follow-up and additional cases are needed to determine the specificity, prevalence, and clinical relevance of these bladder changes in JEB.

## Conclusion

This report describes a novel bladder phenotype in a patient with junctional epidermolysis bullosa (JEB) carrying compound heterozygous ITGB4 mutations, characterized by focal papillomatous bladder wall thickening and follicular mucosal changes. These findings broaden the recognized phenotypic spectrum of JEB and emphasize the importance of comprehensive urological assessment in patients presenting with urinary symptoms or unexplained bladder abnormalities. Early recognition and targeted intervention may help prevent chronic urological complications. Further investigation into the underlying molecular mechanisms may inform the development of future therapeutic strategies.

## Data Availability

The data underlying this article are available in the article itself; further information can be obtained from the corresponding author on reasonable request.
