# Supplementary figures and images for: A novel bladder phenotype in junctional epidermolysis bullosa: a case report
Source: Front Pediatr. 2025 Aug 21;13:1555599. doi: 10.3389/fped.2025.1555599 (PMC12408581; doi:10.3389/fped.2025.1555599)

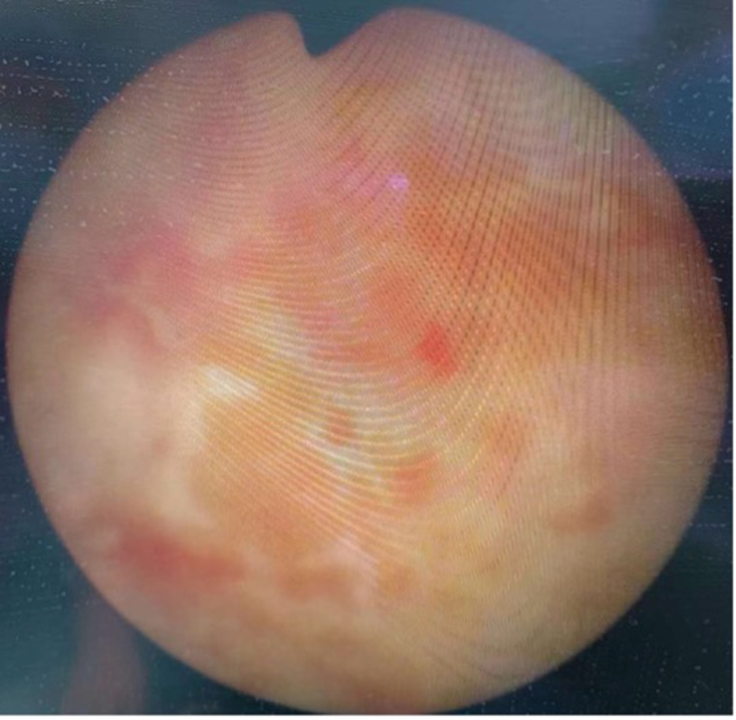

Supplement: Supplementary file 1 [file Image1.jpeg]

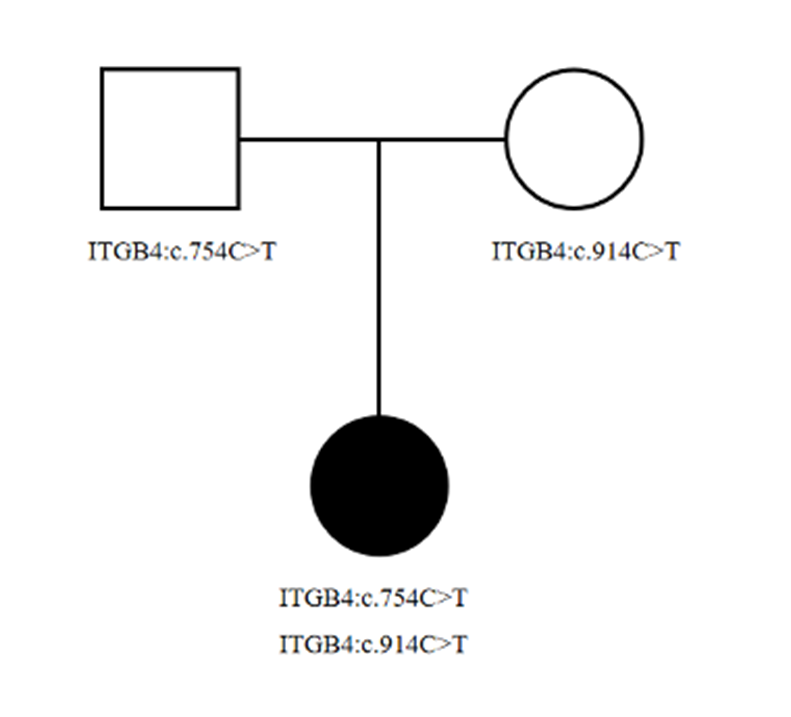

Supplement: Supplementary file 2 [file Image2.tif]

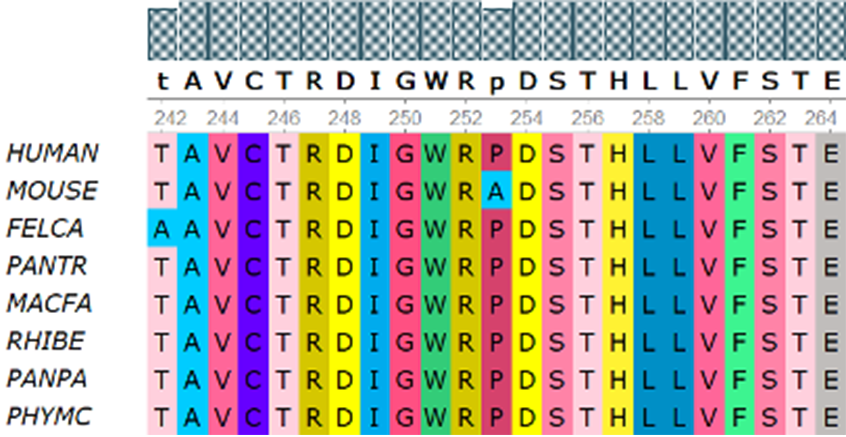

Supplement: Supplementary file 3 [file Image3.tif]

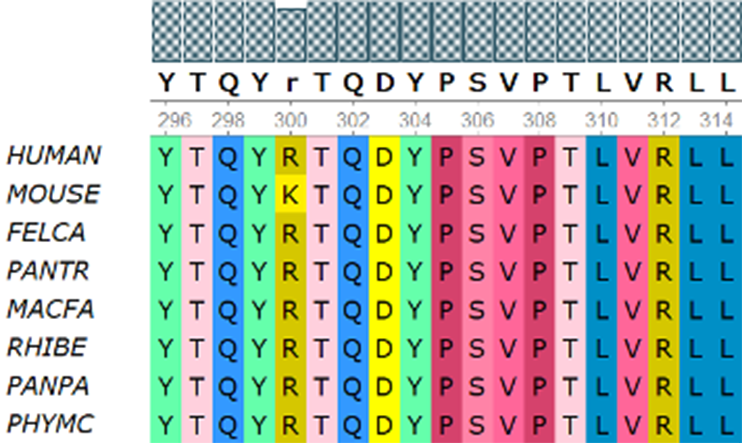

Supplement: Supplementary file 4 [file Image4.tif]
